# Glycemic index and insulin index after a standard carbohydrate meal consumed with live kombucha: A randomised, placebo-controlled, crossover trial

**DOI:** 10.3389/fnut.2023.1036717

**Published:** 2023-02-17

**Authors:** Fiona S. Atkinson, Marc Cohen, Karen Lau, Jennie C. Brand-Miller

**Affiliations:** ^1^School of Life and Environmental Sciences and Charles Perkins Centre, The University of Sydney, Sydney, NSW, Australia; ^2^Extreme Wellness Institute, Melbourne, VIC, Australia

**Keywords:** kombucha, glycemic index, insulin, glucose control, fermentation

## Abstract

**Introduction:**

Kombucha is a complex probiotic beverage made from fermented tea, yet despite extensive historical, anecdotal, and *in-vivo* evidence for its health benefits, no controlled trials have been published on its effect on humans.

**Methods:**

We conducted a randomised placebo-controlled, cross-over study that examined the Glycemic Index (GI) and Insulin Index (II) responses after a standardised high-GI meal consumed with three different test beverages (soda water, diet lemonade soft drink and an unpasteurised kombucha) in 11 healthy adults. The study was prospectively registered with the Australian New Zealand Clinical Trials Registry (anzctr.org.au: 12620000460909). Soda water was used as the control beverage. GI or II values were calculated by expressing the 2-h blood glucose or insulin response as a percentage of the response produced by 50 g of glucose dissolved in water.

**Results:**

There was no statistically significant difference in GI or II between the standard meal consumed with soda water (GI: 86 and II: 85) or diet soft drink (GI: 84 and II: 81, (*p* = 0.929 for GI and *p* = 0.374 for II). In contrast, when kombucha was consumed there was a clinically significant reduction in GI and II (GI: 68, *p* = 0.041 and II: 70, *p* = 0.041) compared to the meal consumed with soda water.

**Discussion:**

These results suggest live kombucha can produce reductions in acute postprandial hyperglycemia. Further studies examining the mechanisms and potential therapeutic benefits of kombucha are warranted.

## 1. Introduction

Kombucha is a beverage made from fermented tea that contains a complex mixture of bacteria and yeast along with a cocktail of organic acids, polyphenols, ethanol, amino acids, and various vitamins and essential elements. Kombucha is growing in popularity due to interest in the human microbiome and purported health benefits that include improvements in blood glucose, cholesterol, and blood pressure readings, and enhanced immune, liver and gastrointestinal function (1, 2). While there is a wealth of historical and anecdotal evidence to suggest kombucha is beneficial to human health, direct research evidence is lacking (2, 3). There are no published controlled clinical trials of kombucha and to date the only published human study of kombucha is a small uncontrolled study that revealed regular daily consumption of kombucha normalised blood glucose values in subjects with non–insulin-dependent diabetes mellitus (4).

While human data is lacking, the effect of kombucha on glucose control is well documented in animal studies. Controlled animal trials report that kombucha reduces blood glucose levels, improves lipid profiles and supports pancreatic, renal and liver function (5–7). The mechanisms of action for these effects are unclear and are likely to occur through multiple processes that include improvements in gut microbiota and islet beta cell function, inhibition of inflammation and insulin resistance, and reduced damage to the intestinal barrier (8). Recent systematic reviews and meta-analyses on the effects of vinegar on postprandial glucose and insulin levels further suggest the acetic and gluconic acid content of kombucha contribute to its effects (9–12).

The rate at which carbohydrate is digested and released into the bloodstream is influenced by many factors, such as the food’s physical form, its fat, protein and fibre content, and the chemical structure of its carbohydrate (13). Over three decades of research has confirmed that similar foods within the same food group can have quite different effects on blood glucose levels and therefore a food’s glycemic effect cannot be accurately predicted solely from the type and amount of carbohydrate it contains. Similarly, postprandial insulin responses of foods cannot always be predicted according to the extent to which they increase blood glucose levels (14–16). The glycemic index (GI) ranks equal available carbohydrate portions of different foods based on the extent to which they increase blood glucose levels after being eaten (17), and the Insulin Index (II) was developed to measure the postprandial insulin response in those same test portions (15). However, most GI studies to date have not concurrently measured glycemic response alongside postprandial insulin response. The aim of this study was to determine the GI and II responses when a standard high carbohydrate, high GI meal is consumed with a complex living kombucha, compared to either soda water or diet soft drink.

## 2. Materials and methods

This study used a randomised, single-blinded, placebo-controlled crossover design based on the internationally standardised GI testing methodology (18). The Human Research Ethics Committee of the University of Sydney approved the study protocol (Approval number: 2017/801). Participants provided written, informed consent before starting the experimental phase of the study. The study was prospectively registered with the Australian New Zealand Clinical Trials Registry (ANZCTR: 12620000460909).

### 2.1. Participants

Sample size calculation (90% power) using data from published GI studies indicated 10 or more participants would be required to detect significant differences among the GI and II values of the treatments (18). To allow for a potential outlier GI response to be excluded, 11 healthy adults with normal glucose tolerance and body mass index (BMI), aged between 18 and 45 years were recruited from the Sydney University Glycemic Index Research Service participant database. Exclusion criteria included illness or food allergy, smoking, regular medication usage other than oral contraceptives, over- or underweight, and following a restrictive diet. Participants maintained their usual food, exercise, and lifestyle habits throughout the study duration.

### 2.2. Study treatments and procedures

The reference beverage, an oral glucose solution containing 50 g available carbohydrate, was prepared as 54.9 g Glucodin™ powder (iNova Pharmaceuticals Aust Pty Ltd., NSW, Australia) dissolved in 250 ml water (Table 1). The reference beverage was consumed by each participant on three separate occasions (sessions 1, 4, and 6). In addition, participants also tested three different beverage treatments which were consumed with a standardised, high GI meal. A computer-generated research randomiser program determined the randomised consumption order for each of the three meal-with-beverage treatments. Each meal and beverage treatment was consumed on one occasion, with at least 1 day in between consecutive test sessions.

**Table 1:** Nutritional composition of the reference glucose solution and the three test meals.

Food	Weight (g)	Energy (kJ)	Protein (g)	Fat (g)	Available carbohydrate (g)	Sugar (g)	Fibre (g)
Reference glucose solution	54.9 g glucose 250 mL water	852	0	0	50	50	0
Rice, peas, soy sauce, and soda water	177.1 g meal 330 mL beverage	1,261	8.6	4.7	52.9^*^	1.9	2.9
Rice, peas, soy sauce, and diet lemonade	177.1 g meal 330 mL beverage	1,278	8.6	4.7	52.9^*^	1.9	2.9
Rice, peas, soy sauce, and kombucha	177.1 g meal 330 mL beverage	1,327	8.6	4.7	55.9^*^^,^^†^	3.6	2.9

* 50 g available carbohydrate provided by the cooked rice, 2.9 g additional available carbohydrate contributed by the green peas and soy sauce.

† 330 ml kombucha beverage contributed an additional 3 g available carbohydrate (1.7 g of which was sugar) to the test meal.

The three beverage treatments were; 330 ml of soda water (Schweppes™, Asahi Beverages, VIC, Australia) that served as a placebo control, diet lemonade soft drink (Schweppes™ Zero Sugar, Asahi Beverages, VIC, Australia), and organic kombucha (The Good Brew Company Pty Ltd., VIC, Australia). The kombucha, which was made from spring water, organic oolong and green tea along with organic sugar, contained a highly complex mix of 200 probiotic species and a high concentration of polyphenols that have been previously characterised (19). The 330 ml of kombucha beverage contributed an additional 3 g of available carbohydrate (1.7 g of which was sugar) to the test meal, while the soda water and diet lemonade did not contain any sugar. The nutritional compositions of the three meal-with-beverage treatments are shown in Table 1. The standardised meal provided 50 g available carbohydrate from microwave Jasmine rice (147.2 g, SunRice™, Ricegrowers Ltd., NSW, Australia), with an additional 2.9 g available carbohydrate provided by green peas (20 g, McCain’s™, McCain Foods Aust. Pty Ltd., VIC, Australia) and soy sauce (10 g, Kikkoman Corporation).

The test portion of microwave Jasmine rice and frozen green peas were combined together in a bowl and cooked in the microwave for 1 min on high. The soy sauce was then added to the prepared meal and immediately served to a participant with the appropriate refrigerated test beverage (soda water, diet soft drink or kombucha). The participants were required to consume all food and fluid served and were instructed to consume the test beverage with the meal (ie. alternate mouthfuls of meal and beverage).

Participants were required to consume a carbohydrate-based evening meal, excluding legumes and alcohol, on the evening prior to each test session. On the morning of each session, participants arrived following a 10–12 h overnight fast. Two capillary blood samples (≥0.5 ml blood) were collected from a warmed hand into heparin-coated tubes in the fasted state (−5 and 0 min). Participants then consumed either the reference glucose solution or one of the test meal-with-beverage treatments within 12 min. Additional capillary blood samples were collected at regular intervals (15, 30, 45, 60, 90, and 120 min) after commencement of the reference solution or test meal. Participants were required to remain seated with minimal movement throughout each 120 min test session.

Each capillary blood sample was centrifuged at 10,000xg for 45 s immediately after collection. The plasma layer was then transferred into an uncoated tube and stored at −30°C for later glucose and insulin analysis. Plasma glucose concentration was measured in duplicate using a glucose hexokinase assay (Beckman Coulter Inc.) on an automatic centrifugal spectrophotometric clinical chemistry analyser (Beckman Coulter AU480^®^, Beckman Instruments Inc., United States). Plasma insulin concentration was measured using an insulin sandwich type enzyme-linked immunoassay (Insulin ELISA kit, ALPCO^®^, Salem, NH, United States). All samples for a given participant were analysed within the same assay.

### 2.3. Data analysis

Incremental area below the 120 min postprandial plasma glucose or plasma insulin response curve (iAUC) was calculated using the trapezoidal rule, ignoring any area below the fasting concentration. Glycemic index (GI) and insulinemic index (II) values for the test meal-with-beverage treatments were determined for each participant by expressing their iAUC response for the test meal relative to their iAUC response to the reference glucose solution (18). Standard non-parametric statistical tests (Wilcoxon signed rank test) were performed using IBM^®^ SPSS^®^ Statistics software (version 28) to assess differences in GI and II values, postprandial glucose and insulin responses, and change from fasting to peak glucose and insulin concentration between the treatments. *p* < 0.05 was considered statistically significant. Results are reported as mean ± standard error of the mean (SEM) unless otherwise stated.

## 3. Results

Eleven healthy adults (4 males and 7 females, mean ± SD age 28.7 ± 4.5 y and mean ± SD BMI 22.6 ± 1.0 kg/m^2^) were screened for eligibility and completed all six test sessions (3 repeated reference glucose solutions and 3 rice with test beverage meals with no missing data points, Supplementary Figure 1). There was no significant difference amongst the mean ± SD consumption times for the three rice-based test meals (soda water: 9:56 ± 1:53 min, diet lemonade soft drink 10:02 ± 1:55 min, and kombucha: 10:04 ± 1:09 min).

### 3.1. Mean glycemic response curves for the reference food and the three test meals

**Figure 1:**
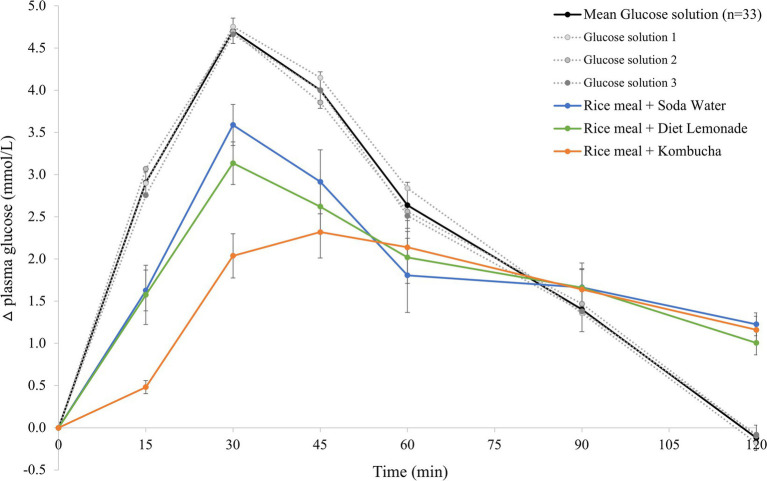
Postprandial glycemic response after test meals. Mean 120 min plasma glucose response curves in 11 healthy participants for the reference glucose solution and the three Jasmine rice-based test meals containing different beverages, shown as the change in plasma glucose from the fasting baseline level. Data are shown as mean ± standard error of the mean (SEM), *n* = 33 for the three repeated glucose solution tests (shown in black, with the three individual glucose solution tests (*n* = 11) shown in grey dotted lines), *n* = 11 for each of the three treatments: soda water meal (shown in blue), diet lemonade soft drink meal (shown in green) and kombucha meal (shown in orange).

The mean 120 min postprandial plasma glucose response curves for the reference glucose solution and the three rice-based meals consumed with different beverages are shown in Figure 1. There were no significant differences in the mean fasting glucose concentrations amongst the reference food and any of the rice-based meals consumed with different beverages (Supplementary Table 1). The reference glucose solution produced the highest peak plasma glucose concentration at 30 min (*p* ≤ 0.006 compared to all test meals) and a larger overall glycemic response (*p* = 0.003 compared to the rice meal with kombucha, *p* = 0.041 compared to the other two meals). The test meals consumed with soda water and diet lemonade soft drink both produced a high peak plasma glucose response at 30 min followed by a steady decline in glycemia between 30 and 120 min. No significant differences were detected between the rice-based test meals containing soda water and diet lemonade. The rice meal containing the kombucha produced a smaller overall glycemic response than the test meal containing soda water (*p* = 0.041). The test meal containing the kombucha produced a steady rise in glycemia to a moderate plateau shaped peak response between 30 and 60 min followed by a gradual decline in plasma glucose concentration between 60 and 120 min. The change in plasma glucose from baseline to peak was significantly lower for the kombucha test meal compared to the test meal containing soda water (*p* = 0.003) and diet lemonade (*p* = 0.008).

### 3.2. Mean plasma insulin response curves for the reference food and three test meals

**Figure 2:**
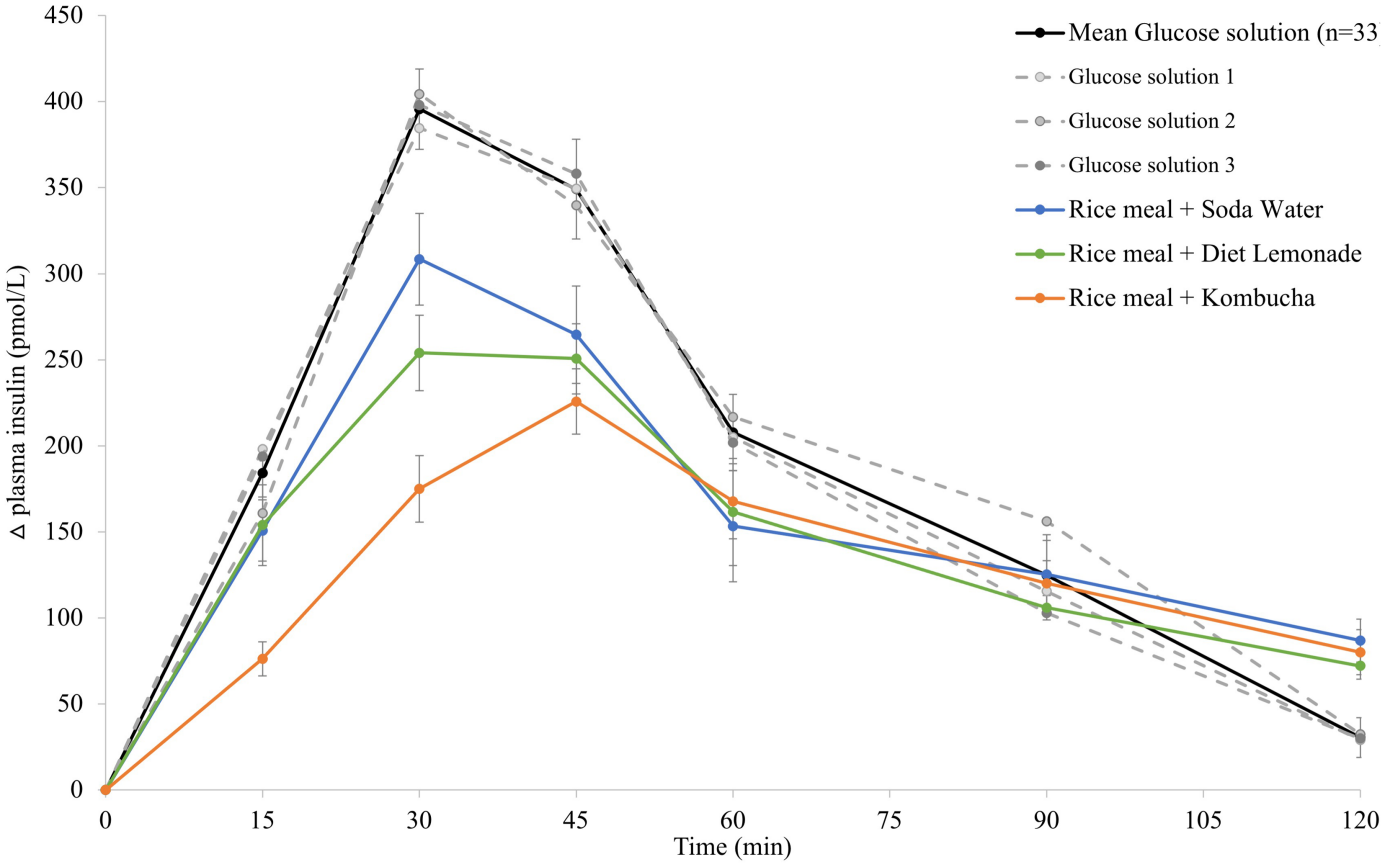
Postprandial insulin response after test meals. Mean 120 min plasma insulin response curves in 11 healthy participants for the reference glucose solution and the three Jasmine rice-based test meals containing different beverages, shown as the change in plasma insulin from the fasting baseline level. Data are shown as mean ± standard error of the mean (SEM), *n* = 33 for the three repeated glucose solution tests (shown in black, with the three individual glucose solution tests (*n* = 11) shown in grey dotted lines), *n* = 11 for each of the three treatments: soda water meal (shown in blue), diet lemonade soft drink meal (shown in green) and kombucha meal (shown in orange).

The mean 120 min postprandial plasma insulin response curves for the reference glucose solution and the three rice-based meals consumed with different beverages are shown in Figure 2. There were no significant differences in the mean fasting plasma insulin concentrations amongst the reference food and any of the rice-based meals consumed with different beverages (Supplementary Table 1). The reference glucose solution produced a rapid initial rise in plasma insulin concentration to the highest peak insulin response at 30 min (*p* = 0.033 compared to the soda water meal and *p* = 0.003 compared to the diet lemonade soft drink or kombucha meals) and a greater overall insulinemic response throughout the experimental period (*p* = 0.003 compared to the kombucha meal, *p* = 0.010 compared to the diet lemonade meal, and *p* = 0.008 compared to the soda water meal). Similar to their glycemic responses, the soda water and diet lemonade test meals produced a high peak plasma insulin response at 30 min whereas the kombucha test meal showed a peak insulin response at 45 min. All three test meals produced a steady decline in insulinemia between 45 and 120 min. The rice meal containing the kombucha produced a smaller overall insulinemic response than the test meal containing soda water (*p* = 0.033).

### 3.3. Glycemic index and insulinemic index values of the test meals

Differences in the total glycemic and insulinemic responses produced by the reference glucose solution and test meals are more clearly reflected by their GI and II values. The reference glucose solution’s GI value (assigned 100) was significantly greater than the mean GI values produced by all three test meals: soda water meal (86 ± 6, *p* = 0.050), diet lemonade meal (84 ± 6, *p* = 0.041), kombucha meal (68 ± 4, *p* = 0.003). The mean GI value produced by the kombucha meal (68 ± 4) was significantly lower than the GI values for the soda water meal (86 ± 6, *p* = 0.041) and the diet lemonade meal (84 ± 6, *p* = 0.050), with an observed reduction equivalent to ~17 absolute GI points lower or ~20% reduction.

The reference glucose solution produced a significantly greater II value (assigned 100) compared to all three test meals: soda water meal (85 ± 4, *p* = 0.008), diet lemonade meal (81 ± 6, *p* = 0.013), kombucha meal (70 ± 4, *p* = 0.013). The II value for the kombucha meal was found to be significantly lower than the II value for the rice-based meal containing soda water (*p* = 0.041). The kombucha meal produced an 11 absolute unit reduction in II value compared to the II value for the Jasmine rice-based meal containing the diet lemonade soft drink, however this difference did not reach statistical significance (*p* = 0.075).

## 4. Discussion

This is the first report of a controlled trial of kombucha in humans and the first study to show that compared to soda water or diet soft drink, a standard serve of kombucha reduces the acute 120 min postprandial glycemic and insulin responses to a high-GI meal. These findings suggest consumption of kombucha with meals could have important health consequences. Long term consumption of high glycemic diets, which induce high and recurrent surges in blood glucose and insulin levels, increase the risk of insulin resistance, dyslipidaemia, and the development of cardiovascular disease, non-insulin-dependent diabetes mellitus and certain cancers (20–26). Conversely, epidemiological and experimental data show that low-GI diets can reduce the risk of these diseases, improve blood glucose control and insulin sensitivity in people with diabetes, reduce high blood fat levels, and can be useful for weight control (20, 22, 23, 27, 28).

The Jasmine rice-based meals consumed with either soda water or diet lemonade were both found to produce high GI values of 86 and 84, respectively. These values are consistent with the GI values of 82 for the same Jasmine rice consumed alone in a 50 g available carbohydrate test portion (The University of Sydney, unpublished data 2019) and 84 when tested in a 25 g available carbohydrate portion (29), showing that the consumption of either of these beverages with a high-GI meal had no significant impact on postprandial glycemia. In comparison, when the kombucha was consumed with the same portion of Jasmine rice, the meal had a GI value of 68, lowering the GI rating of the meal from “high” to “medium”. The kombucha test meal showed the lowest postprandial glucose and insulin responses despite having a slightly higher available carbohydrate content in that meal (55.9 g vs. 52.9 g in the other two test meals, or 50 g in the reference glucose solution).

The mechanisms underlying the beneficial influence of kombucha on the postprandial glucose and insulin responses to the high-GI Jasmine rice meal observed in this study are not clear. The observed delayed and flattened postprandial glucose response with the kombucha beverage suggests it slows down the rate of starch digestion and absorption as previously reported with other fermented foods (30, 31) and vinegar (8–11). However, beverage acidity alone does not appear to explain the results of this study as both the kombucha (pH = 3) and the diet lemonade soft drink (pH = 3.21) (32) had similar, relatively low pH values, yet only the kombucha reduced the postprandial glucose response. The presence of antinutrients, such as tannins in kombucha, which was made from both oolong and green teas, may have also slowed the rate of carbohydrate digestion by binding to the main starch digestion enzyme, alpha-amylase (33). It is also possible that acid-tolerant micro-organisms in the kombucha metabolised some of the glucose in the warm environment of the stomach.

It is likely that multiple mechanisms are in play and that the low pH of kombucha, the complex mix of chemical constituents including high levels of organic acids, polyphenols and tannins, and the actions of live micro-organisms micro-organisms all helped to produce the observed reductions in postprandial glucose and insulin responses. While enhanced postprandial glucose regulation is likely to have many flow-on health benefits, there may be additional benefits from regular kombucha consumption due to changes in the gut microbiota, improvements in islet beta cell function, function or reductions in insulin resistance, inflammation, or damage to the intestinal barrier, which have been observed with regular kombucha consumption in animals (8).

This study used a randomised, single-blinded, placebo-controlled, crossover design along with a research methodology that has been well-established for examining postprandial glycemic and insulinemic responses (18). The robust design along with the use of healthy adults consuming meal and beverage combinations that are consistent with real-world scenarios, makes this research relevant to a wide audience. However, the acute nature of this study, small sample size and lack of clinical context makes it difficult to extrapolate the results to the potential impact of long-term consumption of kombucha or to people with specific diseases. Furthermore, the results cannot be generalised to other kombucha beverages as variation in tea bases, the bacteria and yeast species used as a starter culture, and specific fermentation conditions contribute to large differences in the chemicals, metabolites, microbes, and antioxidant activities of kombucha products (34).

## 5. Conclusion

This study demonstrated a realistic, standard serve of kombucha can produce clinically significant reductions in postprandial glycemia and insulinemia in healthy adults when consumed with a high-GI, rice-based meal. Further studies examining the mechanisms and the potential therapeutic benefits of kombucha on postprandial glycemia in different populations are warranted.

## Data availability statement

The data from this study are available on request to the corresponding author.

## Ethics statement

This study involving human participants was reviewed and approved by the Human Research Ethics Committee of The University of Sydney (Approval number: 2017/801). The participants provided their written informed consent to participate in this study.

## Author contributions

FA, MC, and JB-M: conceptualization. FA and JB-M: methodology. FA and KL: investigation. FA: data curation. FA and MC: writing—original draft preparation. FA, MC, KL, and JB-M: writing—review and editing. MC: funding acquisition. All authors contributed to the article and approved the submitted version.

## Funding

This research was funded by Australia Innovation Connections Grant ICG001289.

## Conflict of interest

FA and JB-M manage The University of Sydney’s glycemic index testing service and are directors of the Glycemic Index Foundation, a not-for-profit health promotion charity. FA and JB-M are authors of books in The New Glucose Revolution series (De Capo, Cambridge, MA). MC is a consultant to The Good Brew Company and co-owner of Extremely Alive Pty Ltd., which produces wellness tonics based on Good Brew Kombucha. He was involved in the design of the study, writing the manuscript and decision to publish the results. He was not involved in the collection, analyses, or interpretation of data.

The remaining author declares that the research was conducted in the absence of any commercial or financial relationships that could be construed as a potential conflict of interest.

## Publisher’s note

All claims expressed in this article are solely those of the authors and do not necessarily represent those of their affiliated organizations, or those of the publisher, the editors and the reviewers. Any product that may be evaluated in this article, or claim that may be made by its manufacturer, is not guaranteed or endorsed by the publisher.
