# Supplementary material for: Glycemic index and insulin index after a standard carbohydrate meal consumed with live kombucha: A randomised, placebo-controlled, crossover trial
Source: Front Nutr. 2023 Feb 17;10:1036717. doi: 10.3389/fnut.2023.1036717 (PMC9982099; doi:10.3389/fnut.2023.1036717)
Supplement: Supplementary file 1 [file Table_1.pdf]

Supplementary Table 1. Mean fasting plasma glucose and insulin concentrations of the reference glucose solution and the three test meals. Data are shown as mean ± standard error of the mean (SEM), n = 33 for the three repeated glucose solution tests and n = 11 for the three test meals.

Treatment	Fasting glucose (mmol/L)	Fasting insulin (pmol/L)
Reference glucose solution	4.95 ± 0.07	32.35 ± 1.55
Rice meal + Soda Water	5.05 ± 0.08	32.99 ± 1.57
Rice meal + Diet Lemonade	5.02 ± 0.08	32.58 ± 2.23
Rice meal + Kombucha	5.02 ± 0.08	33.03 ± 1.86
