# Supplementary material for: Glycemic index and insulin index after a standard carbohydrate meal consumed with live kombucha: A randomised, placebo-controlled, crossover trial
Source: Front Nutr. 2023 Feb 17;10:1036717. doi: 10.3389/fnut.2023.1036717 (PMC9982099; doi:10.3389/fnut.2023.1036717)
Supplement: Supplementary file 2 [file Image_1.pdf]

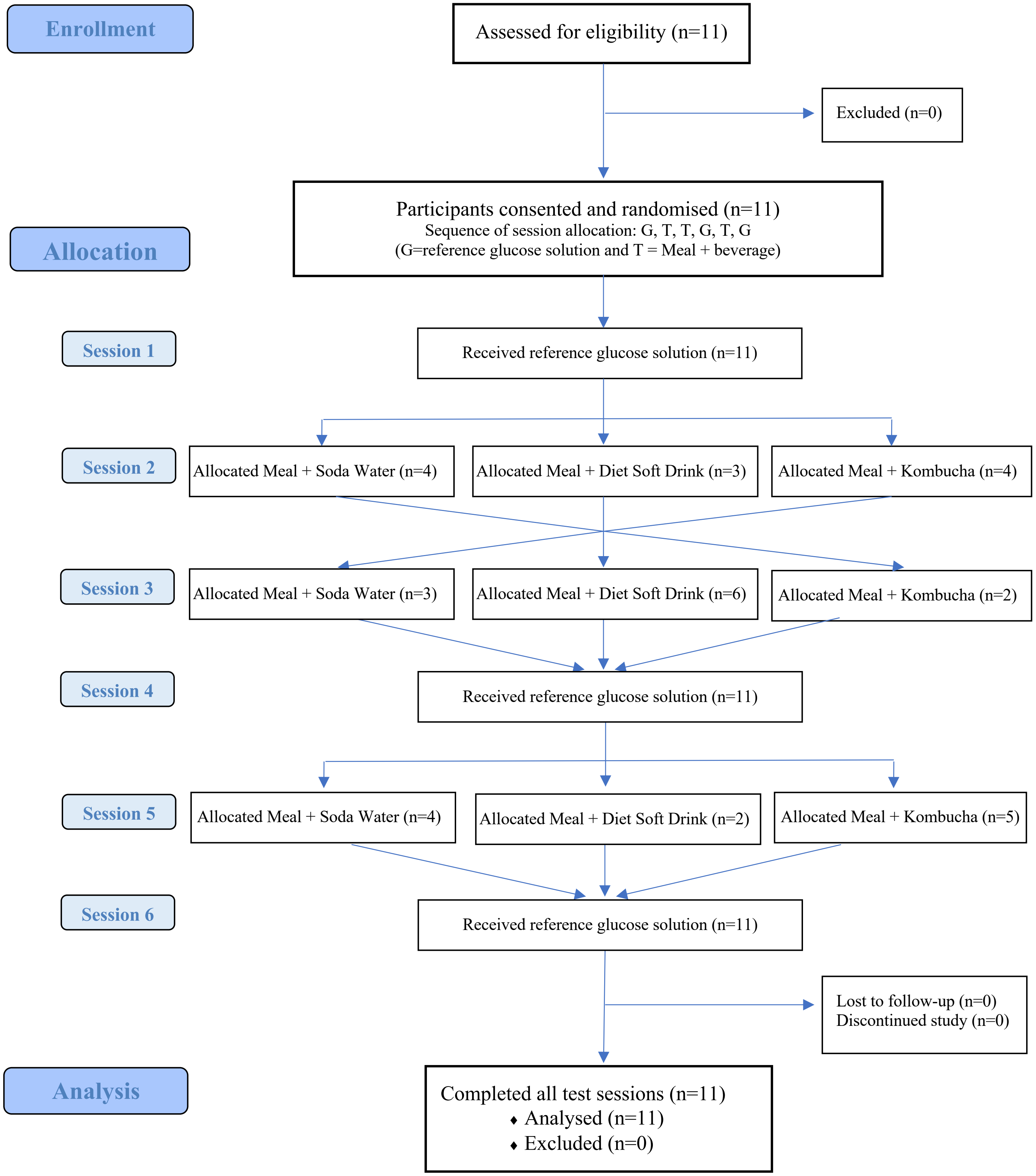

Supplementary Figure 1. Participant progression through the randomised, single-blinded crossover study. The reference glucose beverage was consumed by each participant on three separate occasions (sessions 1, 4, and 6) and a computer-generated research randomiser program determined the randomised consumption order for each of the three meal with beverage treatments.
